# A157 FIT-POSITIVE COLONOSCOPY FINDINGS IN NOVA SCOTIA STRATIFIED BY SEX, RACE, AND REGIONAL POPULATION DENSITY

**DOI:** 10.1093/jcag/gwac036.157

**Published:** 2023-03-07

**Authors:** R Sullivan, J Jones, C Williams, E Kilfoil, D MacIntosh, M Stewart

**Affiliations:** 1 Department of Medicine, Dalhousie University; 2 Colon Cancer Prevention Program, Nova Scotia Health, Halifax, Canada

## Abstract

**Background:**

Population-based colorectal cancer (CRC) screening programs aim to minimize disparities in CRC rates through universal access. However, Canadian CRC mortality rates remain inversely associated with socioeconomic status and rural residence. In the United States some racialized groups have higher rates of advanced adenomas and CRC. Little is known about pre-cancerous findings or CRC mortality amongst racialized groups in Canada because race and ethnicity data are not routinely collected.

**Purpose:**

To determine whether FIT-positive colonoscopy incident adenomas and CRC differ on the basis of sex, race, and regional population density in a provincial CRC screening program.

**Method:**

In this retrospective cohort study drawn from the Nova Scotia Colon Cancer Prevention Program database, we identified adults who had a positive FIT from 2011 to 2021. This report describes incident adenomas and CRC, stratified by sex, race (white vs. racialized groups), and regional population density (urban vs. rural). Racialized groups included those who self-identified as Black/African Canadian, Asian, Middle Eastern and Indigenous. Urban was defined as population centers with more than 5000 individuals. Colonoscopy findings were categorized as no findings, low-risk adenoma (LRA), high-risk adenoma (HRA), or CRC. Comparison between categorical variables was performed with a chi-square test and a t-test for continuous variables. P-value <0.05 was considered significant.

**Result(s):**

41,209 adults (mean age 63.9) had a positive FIT and 34,636 went on to have a colonoscopy offered by the screening program. The FIT-positive colonoscopy participation rate was 84%. Of the 16% overall with a positive FIT but no screening program colonoscopy, 83% had a program consultation but did not proceed with endoscopy for unspecified reasons, 9% declined, and 8.2% are unknown. The overall rate of CRC was 2.4% (n=825) and the adenoma-detection rate was 60.4% (n=20,932). CRC (mean age 65.4) and HRA (mean age 64.6) were associated with older age (p <0.01). Males were more likely to have HRA (38.4% of males) or LRA (26.6% of males) identified compared to females, and females were more likely to have no colonoscopy findings (47.8% of females). CRC was more likely to be identified in urban (2.8%) than rural sub-populations (2.0%). No difference in adenomas or CRC incident rates were noted between white and racialized sub-groups.

**Image:**

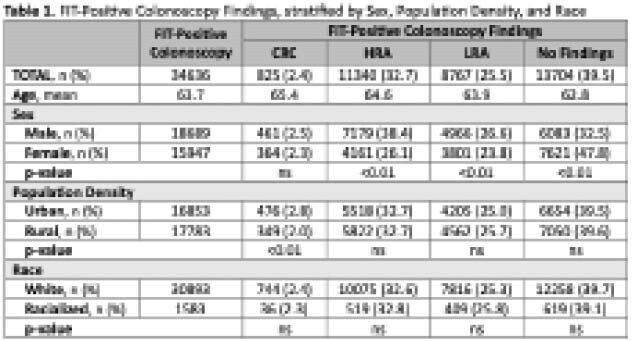

**Conclusion(s):**

This analysis of a provincial CRC screening program suggests that males and urban sub-populations had more high-risk findings during FIT-positive colonoscopies. In the first reported Canadian data, incident rates of adenomas and CRC were similar in white and racialized sub-groups.

**Please acknowledge all funding agencies by checking the applicable boxes below:**

None

**Disclosure of Interest:**

None Declared

